# Mental health in Para athletes—interaction with physical health problems in prospective monitoring

**DOI:** 10.3389/fpsyg.2025.1628494

**Published:** 2025-07-09

**Authors:** Aglaja Busch, Verena Meidl, Rainer Leonhart, Berit Bretthauer, Petra Dallmann, Eva Johanna Kubosch, Anja Hirschmüller

**Affiliations:** ^1^Bern Movement Lab, Division of Physiotherapy, School of Health Professions, Bern University of Applied Sciences, Bern, Switzerland; ^2^Department of Orthopedics and Trauma Surgery, Medical Center – Albert-Ludwigs-University of Freiburg, Freiburg im Breisgau, Germany; ^3^Department of Psychology, Albert-Ludwigs-University of Freiburg, Freiburg im Breisgau, Germany; ^4^Libermenta Hospital, Freudental, Germany; ^5^ALTIUS Swiss Sportmed Center AG, Rheinfelden, Switzerland

**Keywords:** mood, stress, PHQ-4, regression tree, injury, illness

## Abstract

**Background:**

Evaluation of health problems in the Para athlete cohort is well-established. Nonetheless, analyses of the association between mental health and injury, illness or variability of training and competition are spare. Therefore, the purpose of this prospective observational study was to assess this potential relationship in a cohort of Para athletes.

**Methods:**

Continuous health monitoring of German Paralympic athletes using the Oslo Sports Trauma Centre (OSTRC) questionnaire and Patient Health Questionnaire-4 (PHQ-4) on a weekly basis. Additionally, primary sporting activity, training exposure, and subjective training intensity per week were recorded. PHQ-4 scores in relation to substantial health problems were analyzed [mean (M) and 95% confidence interval (95%CI)]. A regression tree analysis was used to analyze the relationship between the independent variables age, sex, impairment type, and sport, as well as the stress level, mood, PHQ-4 sum score, subjective training intensity, training exposure, main weekly activeness, type of health problem, and if it was a substantial health problem, 4 consecutive weeks and dependent variable, the PHQ-4 sum score in the fifth week.

**Results:**

Over an observation period of 124 weeks, 122 Para athletes reported a total of 438 health problems and a mean PHQ-4 score of *M* = 1.3 (95%CI: 1.3–1.4). Highest mean score was observed during illnesses (M = 2.6; 95%CI: 2.2–3). The regression tree identified the leading PHQ-4 score and current mood or stress level as the primary predictors, while all other independent variables did not contribute to the model's prediction.

**Conclusion:**

The findings suggest a potential impact of physical health concerns on mental health, though these, and variations in training or competition were not identified as predictors for the mental health status in a Para athlete cohort. In addition, mean PHQ-4 scores remained below clinical cut-off values, suggesting the need for individualized support to ensure adequate management.

## 1 Introduction

In recent years, Paralympic sport has gained increasing interest among the general population and in professionalism of the Para athletes. Therefore, research in Para athletes covering the surveillance of injuries and illnesses was well implemented at the Paralympic Games and longitudinal data have been presented recently (Fagher et al., 2020; Busch et al., 2021, 2025; Hirschmüller et al., 2021; Steffen et al., 2022; Derman et al., 2023b,a). A systematic review reported an injury prevalence ranging from 3 to 80% and incidence from 0.1 to 91.0 injuries per 1,000 athlete days, depending on the sports category, demonstrating a large variance across the different participant cohorts. Illness ranged between 5% and 24% with incidences from 3 to 20 illnesses per 1,000 athlete days (Luijten et al., 2024).

Besides injury and illness, the athletes' health and performance might be affected by their mental health condition. Lately, athletes' mental health increasingly moved into the focus of research in the field (Reardon et al., 2019). The prevalence of mental health symptoms was reported to range from 19 to 34% in current elite athletes (Gouttebarge et al., 2019). It is not only thought that generic but also handicap- and sport-specific aspects, such as suffering from decreased performance or severe injury increase the risk of mental health symptoms (Reardon et al., 2019). Mental ill-health with emotional responses like sadness and depression might be elicited by a severe injury or illness and its recovery thereof (Gulliver et al., 2015). Conversely, psychological and sociocultural factors pose potential risks for injuries in sports, too (Reardon et al., 2019). Distress, excessive training behaviors or diminished vitality are discussed to increase injury risk (Wiese-Bjornstal, 2010).

Despite the already introduced mutual interaction of injury or illness and mental health, prospective data in a paralympic cohort is sparse (Reardon et al., 2019). There is limited longitudinal data on the associations between mental health and injuries and illnesses in athletes and Para athletes over the course of an athletic year (Busch et al., 2022; Poucher et al., 2022; Fagher et al., 2023; Meidl et al., 2024b,a). Studies examining the mental health of Para-athletes found a weekly prevalence of 15–30.4% of anxiety/depression symptoms, paired with associations to reported injuries or illnesses (Fagher et al., 2023; Bentzen et al., 2025). Nevertheless, further investigation into the occurrence and association of mental health symptoms and injuries or illnesses throughout seasonal changes in training, competition and regeneration might give valuable information to Para athletes and medical staff.

Therefore, the purpose of this study was to examine data concerning mental health in a cohort of Para athletes, in relation to reported injuries and illnesses, as well as variability in training and competition and to identify predictors for future mental health problems.

## 2 Methods

### 2.1 Participants and data collection

This prospective cohort study started with the identification and subsequent contact of the candidate Para athletes preparing for the next Paralympic Summer or Winter Games in November 2018 through the National Paralympic Committee. The recruitment process as well as design and data collection are described in detail elsewhere (Kubosch et al., 2017; Busch et al., 2021; Hirschmüller et al., 2021). Recording of the data was executed between May 2019 and September 2021. Thus, data recording extended into the COVID-19 pandemic. The present study does not particularly analyze this period. Nonetheless, possible implications are considered in the discussion. Baseline characteristics of participating Para athletes were obtained at the first measurement time point including demographic information, impairment type, according to the IOC Para Translation (Derman et al., 2021), and sport execution [ambulatory (including para swimming) or wheelchair]. The survey consisted of two self-reported weekly online questionnaires (Athlete Monitoring, FitsStats Inc, Canada). First, the Oslo Sports Trauma Research Center questionnaire on new and ongoing health problems (OSTRC-HP) (Hirschmüller et al., 2017; Clarsen et al., 2020) was distributed. Secondly, the participants completed the Patient Health Questionnaire-4 (PHQ-4). A questionnaire screening for depressive and anxiety symptoms (Kroenke et al., 2009) and recently validated for longitudinal mental health surveillance (Meidl et al., 2024a). Additionally, training exposure in hours per week, subjective training intensity on a four-point Likert scale ranging from low/rest to higher than normal and the subjective stress level and mood were recorded. Moreover, the participants were asked to classify their main weekly activeness as either regeneration, training, training camp, competition, or a combination of these, e.g., week of training and competition. This classification was subjectively chosen by the Para athletes and should reflect the major component of the weekly sporting activity.

All participating Para athletes gave their informed consent. The study was pre-registered (DRKS: 00015771) and executed according to the STROBE (Strengthening the Reporting of Observational Studies in Epidemiology) guidelines. Ethical approval was given by the institutional ethics committee (University Freiburg: 254/18) and it followed the Declaration of Helsinki.

### 2.2 Definition and classification of injury, illness, and mental health problems

The criteria for defining injury and illness paralleled established literature. Injuries included disorders of the musculoskeletal system and concussions. These injuries were further classified into two types: acute injuries, which result from a specific injury event, and overuse injuries, which are not associated with a specific event. Disorders affecting other body systems were categorized as illnesses (Bahr et al., 2020; Derman et al., 2021). Health problems (HP) were diagnosed by medical personnel and categorized using the Sports Medicine Diagnostic Coding System (Meeuwisse and Wiley, 2007), administered by a qualified study nurse. Additionally, the classification of HP into substantial and non-substantial was decided upon severity (Clarsen et al., 2020). A health problem was classified as substantial if it caused a moderate to severe reduction in sports performance or training or resulted in complete absence from sport participation (Clarsen et al., 2020).

Classification of PHQ-4 sum scores was: no/minimal symptoms (0–2); mild symptoms (3–5); moderate symptoms (6–8); severe symptoms (9–12) (Kroenke et al., 2009).

### 2.3 Statistical analyses

In case of multiple health problems being reported in the same week, e.g., acute injury and illness, the health problem with the higher severity score was kept in the dataset to increase data correctness, without doubled PHQ-4 scores for the same week. In case of same severity scores, the first reported health problem was chosen. Descriptive statistics of the PHQ-4 sum score (mean, standard deviation, and 95% confidence intervals [95% CI]) were calculated overall and for weeks with substantial health problems using R [V.4.1.0 (R Core Team, 2023)]. Moreover, a regression tree analysis using the Chi-squared Automatic Interaction Detection (CHAID) method using SPSS (version 30.0) was performed to investigate the relationship between the PHQ-4 sum score (dependent variable) and the following independent (predictor) variables: Age, sex, impairment type, and sport. Further independent variables included the stress level, mood, PHQ-4 sum score, subjective training intensity, training exposure, main weekly activeness. Moreover, the presence or absence of a health problem, the type of health problem (if any), and whether the health problem is considered as substantial or not. These variables were recorded on 4 consecutive weeks prior to the PHQ-4 assessment in the fifth week. For this purpose, the data was divided into 5-week sections (bins). The values of the independent variables from the first 4 weeks were integrated into the model to assess their influence on the PHQ-4 in the fifth week. This process was repetitively done for all 5-week sections per participant. The CHAID method divides the dataset into homogenous subgroups by identifying the most significant independent variable based on the chi-squared statistic. Meaning that the independent variable having the highest dependency is accepted. After the initial node, the sample is divided into two subsamples. In each of these, the best predictor is again sought independently for the next steps. Once no statistical significance is found for one node it is the terminal node. In contrast to other prediction models, the different nodes contain specific, disparate predictors. The aim is to identify the final nodes that exhibit the greatest degree of homogeneity. The algorithm can handle non-linear relationships and interactions between predictors without requiring parametric assumptions (Ritschard, 2013; Milanović and Stamenković, 2016). A minimum of 100 cases per superior and 50 cases per inferior node were predefined to prevent overfitting. Significance levels at each node were Bonferroni corrected.

## 3 Results

A total of 124 Para athletes (of approximately 250 invited Para athletes) agreed to participate. Two participants withdrew their consent, leaving 122 participants (mean age: 28 years, age range: 16–61 years) to be included in the analyses (Table 1). The mean participation duration for Para athletes was 63 weeks, with a range of 3–124 weeks. The overall weekly response rate was 85%, ranging from 65% to 100%. Details on participant numbers and response rates can be found in the Supplementary material.

**Table 1:** Participant characteristics.

		***n***	**%**
Sex	Male	64	52.4
	Female	58	47.5
Impairment	Spinal cord related disorders	42	34.4
	Other musculoskeletal impairments^*^	23	18.9
	Limb deficiency	17	13.9
	Other neurological impairments†	16	13.1
	Visual impairment	15	12.3
	Cerebral palsy	6	4.9
	Intellectual impairment	2	1.6
	Other	1	0.8
Sport category	Ambulatory	62	50.8
	Wheelchair	60	49.2
Competitive season	Summer	113	92.6
	Winter	9	7.4
Type of sport	Wheelchair basketball	32	26.2
	Paracycling	18	14.8
	Athletics	13	10.7
	Table tennis	9	7.4
	Swimming	8	6.6
	Wheelchair rugby	7	5.7
	Goalball	6	4.9
	Nordic skiing	6	4.9
	Equestrian	5	4.1
	Rowing	3	2.5
	Shooting	3	2.5
	Judo	2	1.6
	Alpine skiing	2	1.6
	Wheelchair tennis	2	1.6
	Wheelchair fencing	1	0.8
	Sitting volleyball	1	0.8
	Wheelchair curling	1	0.8
	Triathlon	1	0.8
	Canoe	1	0.8
	Boccia	1	0.8

*n*, Number; ^*^for example, leg length differences; ^†^for example, brain disorders.

In total 438 injuries and illnesses of which 284 (64.8%) were classified as substantial were reported by 97 athletes. An equal quantity of illnesses (*n* = 224; 51%) and injuries (*n* = 214; 49%), subdivided into acute injuries (*n* = 115; 26.3%) and overuse injuries (*n* = 99; 22.6%) was present. Details on incidence, time-loss, and burden per illness and injury as well as information on the corresponding region and diagnosis have been published elsewhere (Busch et al., 2025).

Overall, mean PHQ-4 sum scores are displayed in Table 2. Scores were lowest in weeks without reported health problem (1.1; 95% CI: 1.1–1.2) and highest in weeks with a reported substantial illness (2.6; 95% CI: 2.2–3.0).

**Table 2:** PHQ-4 sum score overall and at weeks without reported health problem, with substantial health problem and subdivided into substantial illness, acute injury or overuse injury.

**Condition**	***N* (%)**	**PHQ-4 mean ±SD**	**95% CI**
Overall	7,578 (100%)	1.3 ± 2.1	1.3–1.4
Without health problem	6,353 (83%)	1.1 ± 1.8	1.1–1.2
All sub. health problems	275 (3.6%)	2.4 ± 2.7	2.1–2.8
Sub. illness	158 (2.1%)	2.6 ± 2.6	2.2–3.0
Sub. acute injury	71 (1.0%)	2.1 ± 2.5	1.6–2.7
Sub. overuse injury	46 (0.6%)	2.3 ± 3.3	1.4–3.3

Cave: non-substantial health problems are not considered in the table, resulting in a deviation from the overall number. CI, Confidence interval; SD, standard deviation; sub substantial.

**Figure 1:**
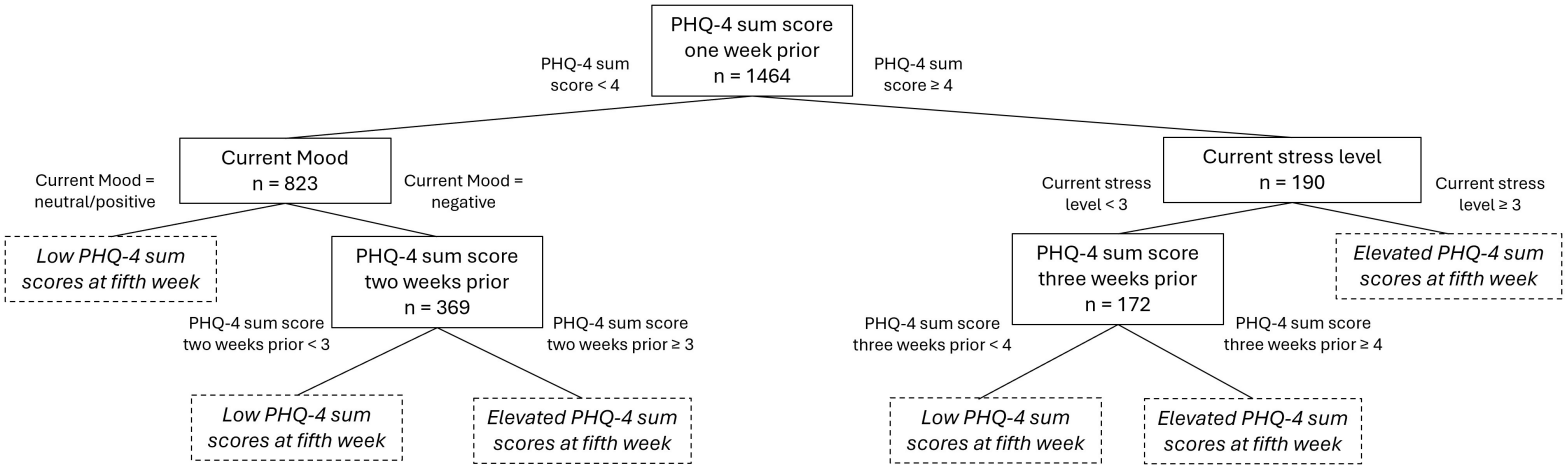
Basic overview of a regression tree displaying the most influential predictors for the PHQ-4 sum score with case numbers and split thresholds. PHQ-4, patient health questionnaire 4; n, case numbers.

A regression tree model was used to examine the influence of various demographical, psychological, and training variables as well as occurrence of illnesses, acute, and overuse injuries with their severity over a 4-week period on the PHQ-4 sum score at the fifth week. The final regression tree comprised 24 nodes, with 15 terminal nodes and a depth of 3. In total 6 variables were identified to be significant predictors for the PHQ-4 in the fifth week. The PHQ-4 score 1 week prior was the most influential predictor (*p* < 0.001). The threshold for the split was found to be at PHQ-4 sums score of 4. This means participants with a lower sum score below 4 one week prior were generally predicted to have a lower PHQ-4 score at the fifth week. Subsequent splits including the current mood (*p* < 0.001), current stress level (*p* = 0.002) and the PHQ-4 score 2 weeks prior to the targeted PHQ-4 (*p* < 0.001). A current negative mood and high stress levels as well as elevated PHQ-4 scores (≥ 3) two weeks prior were found to predicted higher PHQ-4 weeks in the fifth week. The third split demonstrated that higher PHQ-4 3 (*p* < 0.0001) and 4 weeks (*p* = 0.02) prior predict slightly elevated PHQ-4 values in the fifth week. A basic illustration of a regression tree is presented in Figure 1, all results can be found in the Supplementary material. The variables age, sex, impairment type, sport, as well as occurrence and severity of a health problem, training exposure, subjective intensity, and main weekly activeness in 1–4 weeks prior were not considered, meaning to have no predicting influence on the PHQ-4 in our model.

## 4 Discussion

This is the first study investigating the PHQ-4 in relation to parameters describing the psychological, training, and seasonal fluctuation over an observation time of 124 weeks in a Para athlete cohort. Overall, mean PHQ-4 sum scores were low, reflecting no or minimal symptoms but demonstrated elevations in weeks with reported substantial illnesses. Recent and cumulative psychological distress as measured by PHQ-4 sum score in the preceding weeks along with current mood and stress levels were identified as key determinants of mental health status.

The analysis of the PHQ-4 sum scores demonstrated an elevation in correspondence with the reporting of respective substantial injuries or illnesses. The finding aligns with the results of another study, which reported small to medium effect sizes of odds ratios favoring elevated PHQ-4 sum scores in conjunction with these physical health problems in a Para athlete cohort (Bentzen et al., 2025). The PHQ-4 sum scores were considerably elevated during the presence of a substantial illness in the present study. As discussed by Bentzen et al., the uncertainty surrounding, the severity or duration of an illness has the potential to influence mental health outcomes (Bentzen et al., 2025). A similar situation may be observed in the context of overuse injuries. The present study demonstrated that the highest upper limit of the 95% CI of the PHQ-4 sum scores was found in interaction with a reported overuse injury. The potential for recurring and fluctuating symptoms or pain, associated with a gradual onset of overuse injuries, may have a negative effect on mental health. Although the increases in PHQ-4 sum scores during substantial health problems were identified, the 95% CI remained below the score of 4. These values are below the cut-off value for a positive screening, defined in a study validating the PHQ-4 in a Para athlete cohort and focusing on a preventive approach (Meidl et al., 2024a), and did not reach the threshold of ≥ 6 which is considered a yellow flag in a clinical setting (Löwe et al., 2010). Accordingly, the observed mean elevation might not be clinically relevant. Nonetheless, Para athletes with higher scores in weeks without health problems may be equally affected by a health problem, resulting in an increase of the PHQ-4 sum score. This could indicate that the PHQ-4 sum score of this individual is falling into ranges where an intervention is required, however, this remains speculative.

The elevated PHQ-4 sum scores during substantial physical health problems demonstrated as mean and 95% CI could not be supported by the findings of the regression analysis. The regression analysis revealed that neither the reported injuries or illnesses, nor the training exposure, subjective training intensity, main weekly activity, or the non-modifiable characteristics of age, sex, and impairment contributed to the prediction of the PHQ-4 sum score. Although there had been previous reports of an elevated prevalence of depressive symptoms during the weeks of the Paralympic Games (Bentzen et al., 2022), the present findings did not support this influence of competitions on the PHQ-4 sum score. However, this may be influenced by the assessed competitions in the present study, which could include besides tournaments also, for example, single games in a team sport, which clearly reflects a different setting than the Paralympic Games. It is noteworthy, that sex differences in PHQ-4 sum score have been reported in this cohort (Meidl et al., 2024b). Nonetheless, the present study did not identify sex as a predictor. This finding aligns with the results of latent growth model analysis conducted on a general population sample and a cohort of Para athletes during a period of the COVID-19 pandemic (Busch et al., 2022). It may be postulated, that while differences between the sexes are evident at the group stage, their influence within a global model remains comparatively negligible. Moreover, impairment was not identified as predictor of the PHQ-4 sum score. The group sizes for the individual impairments may be too small to expose a significant influence. The implications of a larger sample size, specifically the potential for contrasting outcomes, are a subject of further discussion. However, it is important to note that achieving this objective may only be feasible in the context of cooperative projects, with the participation of a sufficient number of individuals within each impairment group or Para athlete classification. In this multivariate analysis, the exclusion of subjective training intensity or sex, for example, from the model demonstrated that these predictors are not statistically significant in relation to the included predictors. It is possible that univariate differences exist between the included predictors. The PHQ-4 sum score of the previous weeks, as well as the current mood and stress levels, were identified as the sole predictors. This supports the findings of an examination of the same cohort as presented in this study, which focused on the early identification and follow-up of Para athletes at risk of mental health problems. The study emphasized the implementation of a long-term mental health monitoring strategy to facilitate early identification and rapid response to adverse mental health events (Meidl et al., 2024b). Moreover, the results of the present study identifying the PHQ-4 sum score split value of ≥ 4 for increased PHQ-4 sum scores in the following week further support the aforementioned long-term monitoring approach. The determination of a cut-off value of 4 was made for the purpose of positive screening, with subsequent follow-up by the psychological support (Meidl et al., 2024b). The utilization of this cut-off value should be maintained in accordance with the above-mentioned protocol. Besides the PHQ-4 sum score of ≥ 4, the current mood and current stress level were identified as predictors by the regression model. It is important to note that the feasibility of performing and evaluating the PHQ-4 on a weekly or biweekly basis is contingent upon the available resources of the medical personnel working with Para athletes as well as the Para athletes' compliance in weekly completing a detailed questionnaire. A consecutive checking of the mood or stress level may also be helping to identify mental health problems as demonstrated in the regression analysis. Interestingly, none of the training factors (training exposure and subjective training intensity), the main activity of the week (e.g., training, competition or regeneration), as well as age, sex, impairment or sport were found to be a predictor for the PHQ-4 sum score. It can be discussed that the coping strategies with potential stressful situations, such as training camp with following competition, may be highly individual therefore, confirming an implementation of long-term monitoring. The implementation of a mental health monitoring system could be initiated by the incorporation of a stress level rating as a component of existing assessments of training loads. This can be readily evaluated by coaches and staff working with Para athletes, and it may also assist the athletes themselves in identifying stressful situations. In addition to the provision of monitoring, it is important that information regarding low-threshold assistance, such as national helplines or the contact details of trained staff members within organizations, is made available.

The CHAID regression tree provided a flexible and interpretable model for identifying the key factors influencing the PHQ-4 sum score, offering insights into both immediate and cumulative effects on mental health. Nonetheless, the identified PHQ-4 scores as well as stress level and mood are parameters with high collinearity. This must be kept in mind, when interpreting the data. Moreover, it would be interesting to investigate not only the actual values but the differences in individual PHQ-4 scores over time. For example, if greater change in PHQ-4 sum scores would yield a different prediction in the regression tree model. However, this would be subject to further research. In general, low PHQ-4 sum scores might be indicating a floor effect in this study. More variance in the symptoms of depression or anxiety might also result in a more differentiated pattern of influential predictors.

The present study evaluated the PHQ-4 sum scores as the dependent variable. Nevertheless, it is important to note that a mental health problem may also have a potential impact on physical health or performance during training or competition (Galambos et al., 2005; Rogers et al., 2024). However, a prospective study investigating the mental and physical health of Para athletes over 52 weeks found no association between anxiety/depression and injury or illness in the four leading, current or 4 trailing weeks of the incidence (Fagher et al., 2023). Consequently, the perspective that a mental health problem could also be a contributory factor to other health problems requires further investigation, and it may be interesting to examine this in comparison to Olympic athletes.

The PHQ-4 has been validated and implemented in the monitoring of mental health in Para athlete cohorts (Meidl et al., 2024a,b; Bentzen et al., 2025). However, it is important to acknowledge the limitations of the scale in its capacity to comprehensively capture the full spectrum of mental health symptoms. Its scope is restricted to symptoms of anxiety and depression, leaving out a wide range of other mental health symptoms such as nervousness, eating disorders, alcohol abuse or life satisfaction. It can be hypothesized that physical health problems, training exposure, subjective training intensity and seasonal changes may affect other mental health aspects which are not illustrated here. In order to enhance the inclusion and investigation of further mental health symptoms in elite athletes, the sport mental health assessment tool 1 (SMHAT-1) was developed (Gouttebarge et al., 2021). A longitudinal evaluation with this more comprehensive questionnaire might have yielded other results. Nonetheless, further assessments of the validation and reliability of the SMHAT-1 especially in a Para athlete cohort are needed (Anderson et al., 2023; Waleriańczyk et al., 2025). Possible further steps for practitioners could include the consideration of utilizing advanced assessment tools (e.g., SMHAT-1) to account for various potential mental health problems affecting their athletes. In a subsequent phase, it would be advisable to undertake a bilateral evaluation of the employed tools with the respective athlete, thereby determining the extent of individualized monitoring.

The present study is subject to certain limitations. The cohort under examination consisted of German Para athletes. Cross-cultural variations in the interpretation and expression of mental health symptoms and disorders might be present and need to be considered when compared to other studies. Furthermore, the intensity of the training and the classification of the main activity of the week were reported subjectively by the athletes. Additionally, the content of the training was not further specified. A more structured approach, potentially complemented by data sets such as training records or coach reports, may have led to enhanced accuracy. The PHQ-4 focuses on anxiety and depression, two relevant aspects of the mental health; however, the use of an alternative questionnaire may lead to different results and und thus the generalizability is limited.

The CHAID regression tree is a non-parametric model. It is sensitive to the choice of split points and can be influenced by outliers representing the possibility of overfitting or underfitting. Moreover, out of the longitudinal measurement bins of 5 consecutive weeks have been formed. A variation of this weeks may yield different results. Therefore, the findings should be interpreted in the context of these limitations.

## 5 Conclusion

This study examined the relationship between mental health, as measured by the PHQ-4, and reported injuries and illnesses, in addition to variability in training and competition among a cohort of Para athletes. These results provide valuable insights for mental health professionals, highlighting the necessity of considering both short-term and longer-term psychological factors when assessing and treating individuals at risk of elevated psychological distress. The factors influencing the mental health under investigation appear to be unaffected by age, sex, impairment type, and sport as well as training and seasonal fluctuations in competitions. It is suggested that psychological parameters that are both leading and current may serve as effective predictors. Nevertheless, it is important not to disregard the impact of physical problems or fluctuations in the sporting year on an individual's mental health. A more comprehensive mental health assessment tool might have yielded different results, and its usage should be evaluated in future studies.

## Data Availability

The raw data supporting the conclusions of this article will be made available by the authors, without undue reservation upon reasonable request.
