# Supplementary material for: Mental health in Para athletes—interaction with physical health problems in prospective monitoring
Source: Front Psychol. 2025 Jul 9;16:1628494. doi: 10.3389/fpsyg.2025.1628494 (PMC12283584; doi:10.3389/fpsyg.2025.1628494)
Supplement: Supplementary file 1 [file Data_Sheet_1.pdf]

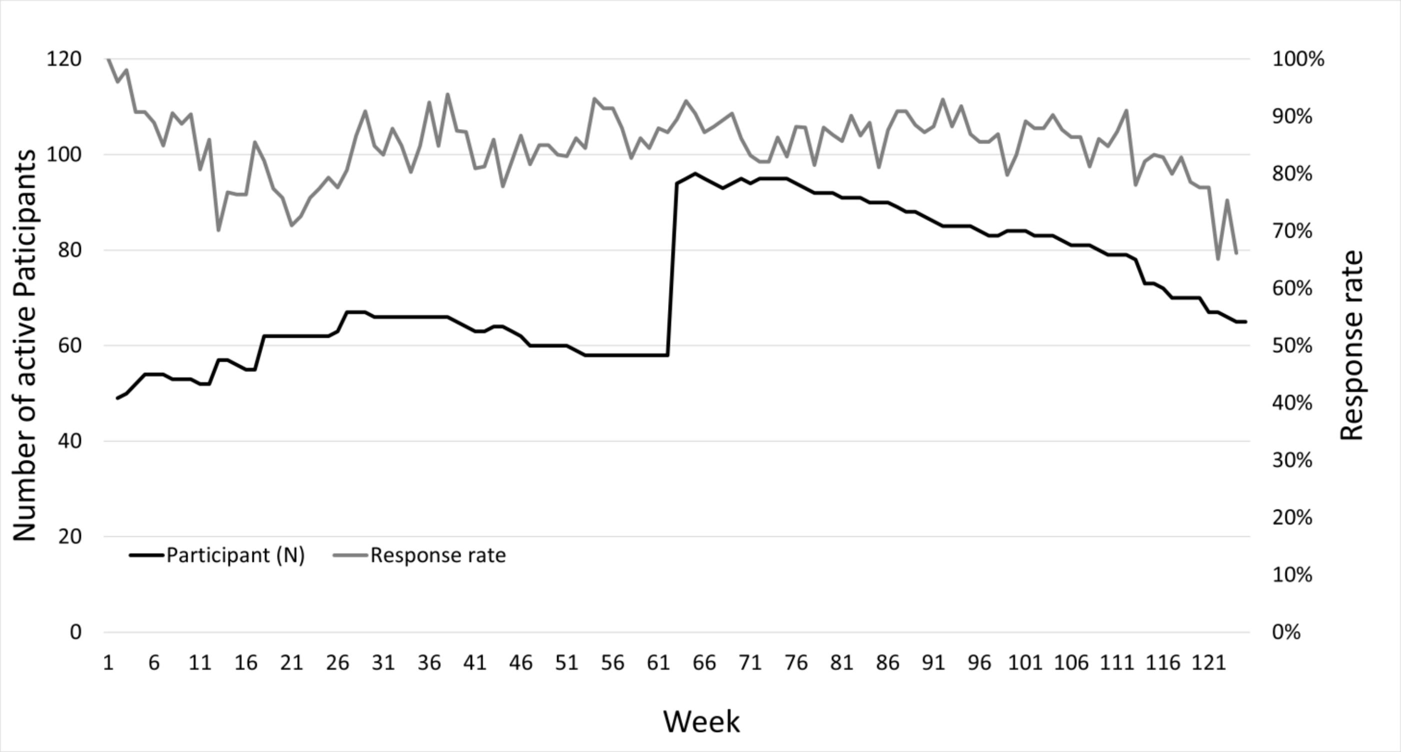

Fig. S1. Number of participants actively corresponding and response rate over 124 weeks

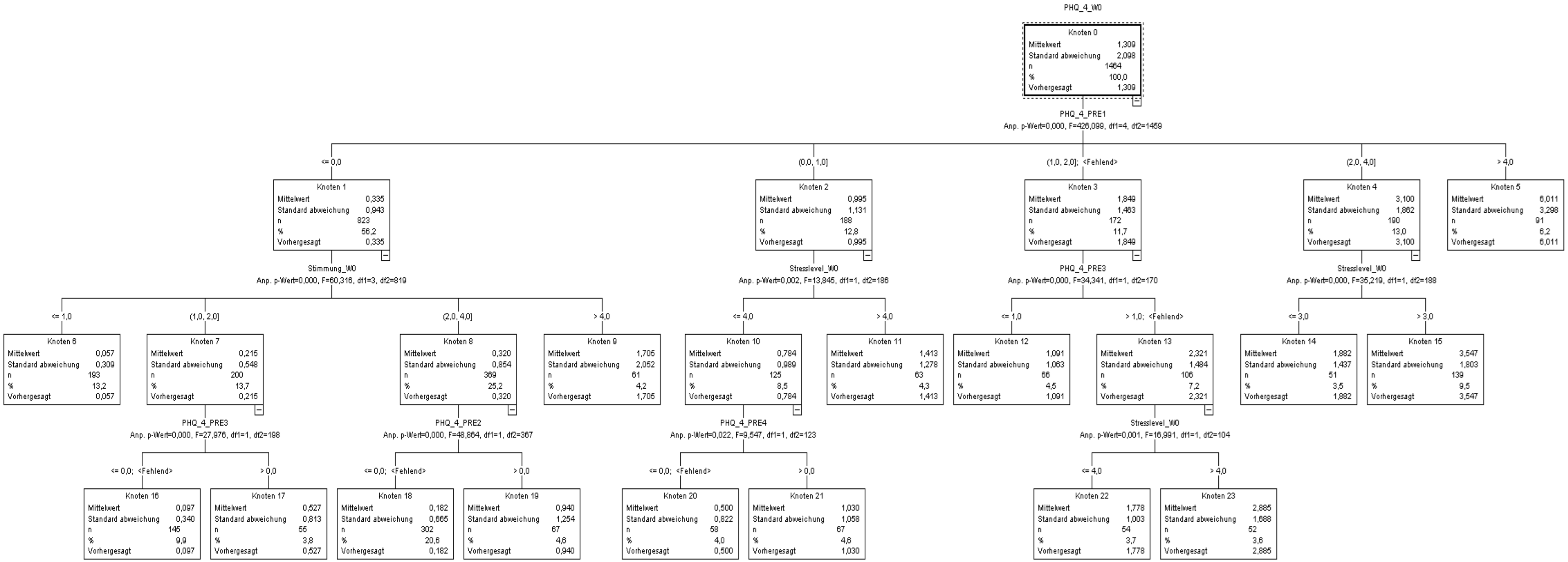

Fig S2. Full output of the regression tree model.
